# Editorial to “An extremely wide QRS complex tachycardia induced by anamorelin”

**DOI:** 10.1002/joa3.13091

**Published:** 2024-06-04

**Authors:** Shujiro Inoue

**Affiliations:** ^1^ Department of Cardiology NHO Kyushu Medical Center Fukuoka Japan

**Keywords:** anamorelin, arrhythmia, sodium channel, wide QRS tachycardia

This is an editorial comment to “An extremely wide QRS complex tachycardia induced by anamorelin.” presented by Shimojo et al.^1^ in the current issue of *Journal of Arrhythmia*.

Cancer cachexia is a multifocal syndrome in patients with cancer characterized by reduced muscle mass and malnutrition, causing progressive functional disability and reduced quality of life. Conventional nutritional support cannot completely reverse cancer cachexia, and useful pharmacologic therapies for cachexia management are limited. Since 2021, anamorelin has been licensed for production and marketing in Japan as a pharmacologic therapy for cancer cachexia. Anamorelin functions as a ghrelin‐like agonist and may stimulate the secretion of growth hormones and appetite by activating the ghrelin receptor, known as growth hormone release promoting factor receptor type 1a (GHS‐R1a). Anamorelin is a drug of interest in the field of cancer cachexia, as several randomized controlled trials have demonstrated efficacy in improving total body weight, lean body mass, quality of life, and appetite in patients with refractory cancer compared with placebo.^2^, ^3^ In all adverse events or serious adverse events, the investigators reported no significant differences in terms of safety.^2^, ^3^ However, this drug exhibited serious side effects, such as conduction disturbance, hyperglycemia, diabetes worsening, and hepatic dysfunction; thus, patient selection and posttreatment monitoring are very important.

Anamorelin generally demonstrates an inhibitory effect on the conduction system because of its Na channel‐blocking properties. Therefore, electrocardiographic abnormalities, atrioventricular block, tachycardia, and bradycardia may appear after anamorelin administration. Additionally, anamorelin is contraindicated in patients with heart failure, ischemic heart disease, severe conduction disturbance, and moderate‐to‐severe hepatic dysfunction. It is administered cautiously to those with a history or risk of QT prolongation and those with conduction disturbances. Periodic electrocardiogram (ECG), pulse, and blood pressure measurements are warranted after anamorelin administration. The mechanism behind anamorelin's proarrhythmic effects remains unknown, but weak binding to sodium channels and L‐type calcium channels was revealed.^4^ Decreased sodium current may predispose to sudden cardiac death, as studies on arrhythmia suppression have demonstrated that sodium channel blockers increase the incidence of sudden cardiac death. Sodium channel blockade‐induced conduction disturbances may cause reentrant arrhythmias because of excitability gap widening.

In the current issue of the *Journal of Arrhytumia*, Shimojo et al. reported a case of drug‐induced wide QRS tachycardia by anamorelin.^1^ Healthcare provider should understand the risk of conduction disturbances and wide QRS tachycardia, although infrequent, as has been reported in several case reports. Anamorelin has increased blood levels in patients with severe hepatic dysfunction. It has been affected by CYP3A4, a hepatic drug‐metabolizing enzyme, and serum concentrations may be increased with drugs that inhibit CYP3A4. Nevertheless, blood concentration alone cannot determine the degree of intoxication, including electrocardiographic abnormalities and proarrhythmias, as factors beyond antiarrhythmic drug concentration, such as electrolytes and genetic factors, also play a role. The appropriate dose was prescribed in all the past wide QRS tachycardia case reports. Some cases may not be predictable because of individual susceptibility to the drug. Some patients respond well to concentrations lower than the general effective blood concentration range, whereas others do not respond even within that range. This is because of the different effective blood sodium channel blocker concentrations required to exert antiarrhythmic effects in individual patients because of genetic differences in myocardial sodium channel function.^5^ In particular, the SCN5A gene encodes the alpha subunit of the cardiac sodium channel Nav1.5 and is responsible for phase 0 of the cardiac action potential. Six related polymorphisms (haplotypes; Hap) are known in the promoter region of the SCN5A gene. Hap B is unique to Asians, with a prevalence of 24% in the Japanese population. Patients who respond to sodium channel blockers at low doses may have Hap B.^5^ Hence, Asians might need to be more observant about anamorelin responsiveness, especially regarding ECG changes early in the administration.

Finally, anamorelin causes potentially fatal arrhythmias and should be carefully monitored before and after administration. The blockade of sodium and L‐type calcium channels mediates the proarrhythmic effects of anamorelin. Sodium channel gene polymorphisms may be associated with pathogenesis in addition to blood levels and dose dependence. Further reports and clarification of the mechanisms are warranted, with the expectation that anamorelin can be used more safely.

## CONFLICT OF INTEREST STATEMENT

The authors declare that they have no known competing financial interests or personal relationships that could have appeared to influence the work reported in this paper.

## FUNDING INFORMATION

None.

## APPROVAL OF THE RESEARCH PROTOCOL

None.

## INFORMED CONSENT

None.

## REGISTRY AND THE REGISTRATION NO. OF THE STUDY/TRIAL

None.

## ANIMAL STUDIES

None.
